# Antiretroviral Treatment-Associated Tuberculosis in a Prospective Cohort of HIV-Infected Patients Starting ART

**DOI:** 10.1155/2011/758350

**Published:** 2010-12-08

**Authors:** William Worodria, Marguerite Massinga-Loembe, Harriet Mayanja-Kizza, Jane Namaganda, Andrew Kambugu, Yukari C. Manabe, Luc Kestens, Robert Colebunders

**Affiliations:** ^1^Department of Medicine, Mulago Hospital, College of Health Sciences, Makerere University, P.O. Box 7051, Kampala, Uganda; ^2^Infectious Diseases Network for Treatment and Research in Africa (INTERACT), Makerere University, Kampala, Uganda; ^3^Infectious Disease Institute, College of Health Sciences, Makerere University, Kampala, Uganda; ^4^University of Antwerp, 2020 Antwerpen, Belgium; ^5^Institute of Tropical Medicine, 2000 Antwerpen, Belgium; ^6^Division of Infectious Diseases, Department of Medicine, Johns Hopkins University School of Medicine, Baltimore, MD, USA

## Abstract

Commencement of antiretroviral treatment (ART) in severely immunosuppressed HIV-infected persons is associated with unmasking of subclinical disease. The subset of patients that are diagnosed with tuberculosis (TB) disease while on ART have been classified as ART-associated TB. Few studies have reported the incidence of ART-associated TB and unmasking TB-IRIS according to the International Network for the Study of HIV-Associated IRIS (INSHI) consensus definition. To determine the incidence and predictors of ART-associated TB, we screened 219 patients commencing ART at the Infectious Diseases Clinic in Kampala, Uganda for TB by symptoms, sputum microscopy, and chest X-rays and followed them for one year. Fourteen (6.4%) patients were diagnosed with TB during followup. Eight (3.8%) patients had ART-associated TB (incidence rate of 4.3 per 100 person years); of these, three patients fulfilled INSHI criteria for unmasking TB-associated IRIS (incidence rate of 1.6 per 100 person years). A body mass index of less than 18.5 kg/m^2^ BMI (HR 5.85 95% CI 1.24–27.46, *P* = .025) and a C-reactive protein greater than 5 mg/L (HR 8.23 95% CI 1.36–38.33, *P* = .020) were risk factors for ART-associated TB at multivariate analysis. In conclusion, with systematic TB screening (including culture and chest X-ray), the incidence of ART-associated TB is relatively low in settings with high HIV and TB prevalence.

## 1. Introduction

Tuberculosis (TB) remains a leading cause of morbidity and mortality in sub-Saharan Africa with the HIV pandemic accounting for 31% of all new TB cases in adults [1, 2]. This is partly because available diagnostic tests are not sensitive enough for early detection of all TB cases [3–5]. Additionally, patients often seek medical care late when they already have advanced HIV disease or are very sick [6–8]. Sputum smear microscopy, the tool for TB screening in most resource-limited settings, has low sensitivity to diagnose TB disease, especially in HIV-infected patients with advanced immunodeficiency [9–11]. 

The World Health Organization (WHO) currently recommends routine screening for TB prior to antiretroviral treatment (ART) initiation [12]. Such screening generally targets symptomatic patients. Therefore, TB patients without symptoms or with atypical symptoms and subclinical disease are often not diagnosed. Intensive screening of HIV-infected patients for TB prior to ART regardless of symptoms has been demonstrated to increase the number of TB cases identified [13, 14]. In a recent study from Durban, South Africa, TB was detected by intensive screening in 158 (19%) of 825 patients undergoing ART preparation [13]. Only 52% of these patients reported cough. It has been demonstrated that patients with subclinical disease started on ART may rapidly progress to symptomatic TB disease as a result of immune reconstitution, and this risk is highest during the first three months of ART [15, 16].

Immune reconstitution inflammatory syndrome (IRIS) is a disorder commonly observed upon ART initiation in severely immune-compromised patients who have concomitant opportunistic infections. HIV/TB coinfection is a leading cause of IRIS. Two clinical types of TB-IRIS have been described: unmasking IRIS (an existing occult/subclinical infection becomes clinically evident after the start of ART) and paradoxical IRIS (worsening of a successfully treated infection following the introduction of ART) [17]. Whereas unmasking IRIS is postulated to result from an imbalanced and exuberant inflammatory response against a viable pathogenic organism in face of a rapidly reconstituting immunity, in paradoxical TB-IRIS, this dysregulated immune response is generally targeted against residual pathogenic antigens. Exact mechanisms for the dysfunctional restoration of pathogen-specific immune responses are not well understood, but defects in antigen-specific activated regulatory and effector CD4 T lymphocytes have been suggested by a number of studies [18–21]. Several studies have reported the incidence of paradoxical TB-IRIS, but relatively few studies have reported the incidence of unmasking and ART-associated TB-IRIS. 

 According to the International Network for the Study of HIV-Associated IRIS (INSHI), ART-associated TB refers to all TB diagnosed during ART, while the subset of patients who develop rapidly progressive signs and symptoms of TB, with exuberant inflammatory features, after initiation of ART are called “unmasking TB-associated IRIS” [22]. In programmes “rolling out” ART, ART-associated TB may be difficult to differentiate from multiple other opportunistic pathologies, thus further delaying diagnosis and appropriate treatment.

 The purpose of this study was to determine the incidence and clinical manifestations of both ART-associated TB and unmasking TB-associated IRIS in an ambulatory HIV care setting in Uganda, where HIV prevalence is high [23]. A secondary objective was to determine the predictive baseline demographic, clinical, and laboratory parameters in patients who develop ART-associated TB.

## 2. Study Methodology

In a prospective cohort of HIV patients eligible for ART at the Infectious Disease Institute (IDI), Kampala, Uganda, we screened 247 patients for study entry. Inclusion criteria were (1) age >18 years, (2) documented HIV infection, (3) ART eligibility according to Uganda Ministry of Health guidelines [24] (CD4 < 250 cells/*μ*L), (4) ART naïve, (5) no evidence of active TB disease by acid fast bacilli (AFB) smear microscopy and chest radiograph (CXR) and (6) willingness to participate in all followup visits and clinical examinations and to have blood drawn for clinical and immunological studies. 

At study enrolment, the HIV serostatus was confirmed using a standard HIV testing algorithm [25], complete blood count with differential CD4 count (FACSCalibur, Becton Dickinson), and C-reactive protein (CRP) (COBAS C-Reactive Protein (Latex), Roche Diagnostics; normal range <5 mg/L) were also measured. Patients were initiated on ART according to the Uganda National ART guidelines [24]. TB screening was based on a standardized symptom questionnaire (presence and duration of cough, chest pain, fever, poor appetite, and weight loss; specifying the duration of each symptom) and physical examination. Patients who had a productive cough of greater than two weeks had sputum collected for TB investigations. Two expectorated sputum samples (early morning) were obtained and sent for microscopy by Ziehl Neelsen (ZN) stain and Fluorescence microscopy (FM); and mycobacterial cultures were performed at the national TB reference laboratory on Lowenstein-Jensen (LJ) culture media. A sputum smear was considered AFB positive if there were at least 10 bacilli per 100 fields. A positive mycobacterial culture was defined as any presence of *Mycobacterium tuberculosis* (*M. tb*) colonies within a period of 6 to 8 weeks of incubation on LJ media. A tuberculin skin test (TST) was administered by intradermal injection of 2TU of RT23 in the volar aspect of the left forearm, and all participants were instructed to come back for its reading 48–72 hours later. A positive TST was defined as a skin induration of at least 5 mm diameter. All patients had CXRs done, and these were examined by a radiologist and reported as normal (unlikely pulmonary tuberculosis (PTB)), possible PTB (infiltrates, nodules, or other abnormality), or probable PTB (with cavitation, adenopathy, pleural effusion, or miliary pattern). Patients judged to have active TB (by clinical assessment, sputum microscopy, or CXR) at ART commencement were started on TB treatment and excluded from the study. Asymptomatic participants with a positive TST and judged to be free of TB based on CXR and sputum culture results were offered isoniazid prophylaxis.

Study participants were followed up and regularly assessed for signs and symptoms suggestive of TB at 2, 4, 8, and 12 weeks after ART initiation, and quarterly thereafter up to one year. Further TB investigations were performed during followup if the patient developed new or worsening symptoms suggestive of TB. This included two sputum microscopy examinations for AFB and culture on LJ media, a CXR (which was compared to one at ART treatment start for evidence of worsening), and an abdominal ultrasound. Patients diagnosed with TB were started on TB treatment and censored from the study. Further routine followup for TB treatment was conducted at the national TB Leprosy programme clinic. The laboratory examinations (haematology, immunology) were performed at the Joint Clinical Research Centre (JCRC) and Makerere University-John Hopkins University (MU-JHU) Core laboratories in Kampala; microscopy examination for AFB and mycobacterial cultures was performed at the National TB Reference Laboratory in Kampala and JCRC TB laboratory.

### 2.1. Study Definitions

TB was diagnosed by a study medical officer according to WHO recommendations [26]. PTB diagnosis was based on at least one sputum AFB-positive result; a positive culture result or an abnormal CXR with a clinical improvement after initiation of TB treatment. For diagnosis of extrapulmonary TB, we considered one sample from an extrapulmonary site which was AFB positive or *M.tb* culture positive, or histological or strong clinical evidence of TB and decision to treat with a full course of TB treatment. Patients who developed signs and symptoms suggestive of TB during followup were evaluated by the study medical officer as TB-IRIS suspects using a standard questionnaire including the INSHI TB-IRIS criteria. Two of the study investigators (W. Worodria and R. Colebunders) later classified these patients as ART-associated TB with or without unmasking TB-IRIS according to the INSHI case definitions (see Table 1) [22]. TB was defined as prevalent if the participant had significant symptoms before the start of ART (a cough of at least two weeks with or without chest pain, fever, or poor appetite) with a positive AFB smear or *M.tb* culture, or radiological abnormality with response to TB, treatment. Patients who had none of the above features at ART initiation but developed them later during the course of treatment were classified as incident TB. As ART-associated TB, we included all patients diagnosed with TB after the start of ART regardless of the fact that after the start of ART a sputum culture obtained before the start of ART was found to be positive. We did not include ART-associated TB patients diagnosed after the start of ART if retrospective review of clinical files indicated that the diagnosis of TB was missed according to WHO guidelines.

### 2.2. Data Management and Statistical Analysis

Patient data was collected on case report forms, monitored by clinical research associates, double entered, validated and stored in an SQL database, and exported to STATA version 10.1 (STATA, College Station, TX, USA) for analysis. Baseline characteristics for patients with prevalent TB, incident TB, and without TB were compared. The predictive value of clinical and laboratory parameters for any diagnosis of TB and specifically for ART-associated TB was examined by Cox proportional hazards and reported as hazard ratios. The time from start of ART to all diagnosis of TB and ART-associated TB was represented as events on a Kaplan Meier curve. BMI was categorized according to WHO recommendations with underweight defined as BMI < 18.5 kg/m^2^ [27].

### 2.3. Ethics

 Ethical approval for the study was obtained from the Infectious Disease Scientific Review Committee, Makerere Faculty of Medicine Ethics Committee, the Uganda National Council on Science and Technology, and the University of Antwerp Ethics Committee.

## 3. Results

**Figure 1:**
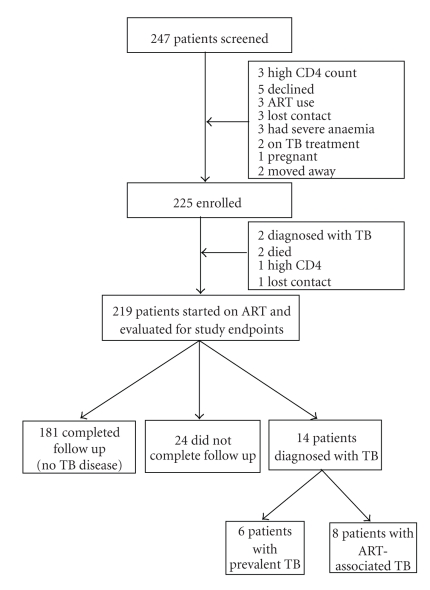
Study enrollment and retention.

Of 247 patients evaluated for ART eligibility, 225 were enrolled, 17 were not eligible, and five were lost to followup (Figure 1). The median age of study participants was 35.7 years (interquartile range (IQR) 30.5–41.3 years) and 157 (70%) were females. The median CD4 count of the study participants was 130 cells/*μ*L (IQR 62–170), and 99 (44%) of the patients were in WHO clinical stage 3 or 4 at enrolment. Sixty eight (30%) of 220 patients had a positive tuberculin skin test at enrolment. The median time from enrolment to ART initiation was 10 days (IQR 7–17). Nine (4.1%) patients who initiated ART died, two patients withdrew consent, and 7 (3.2%) moved out of the study area. Six (2.7%) patients were lost to followup and three were empirically started on TB treatment. Five of the 9 patients who died had CD4 counts less than 50 cells/*μ*L, and these 5 died with clinical signs and symptoms suggestive of sepsis. One patient died of gastroenteritis with severe dehydration; in one patient, the cause of death was not established.

Of all patients screened for TB, 28 (13%) had cough of greater than two weeks' duration. Fifty (23%) patients had at least one symptom (cough, chest pain, fever, poor appetite, or weight loss) for greater than two weeks. Baseline characteristics of patients diagnosed with prevalent TB, incident TB, and without TB are presented in Table 2. Over one year of followup, 14 (6%) patients were diagnosed with TB after starting ART (incidence rate of 6.9 per 100 person years). Of these, 6 (43%) had prevalent TB (2 had positive TB cultures and 4 were sputum AFB smear negative but had abnormalities on CXR) (Table 3). TB in these patients was unrecognized and therefore untreated prior to ART start. The remaining 8 (57%) were considered to be ART-associated TB (incidence rate of 4.3 per 100 person years). Three of these patients (patients 2, 6, and 7 in Table 4) had symptoms consistent with unmasking TB-associated IRIS (incidence rate of 1.6 per 100 person years). In 5 of 8 patients with ART-associated TB and in 2 out of 3 patients with unmasking TB-associated IRIS, TB was diagnosed before an increment in absolute CD4 counts (Table 4). Isoniazid prophylaxis was provided to 23 (34%) of 68 eligible patients with normal CXR and no evidence of TB. There were no complications reported among patients on prophylaxis.

**Figure 2:**
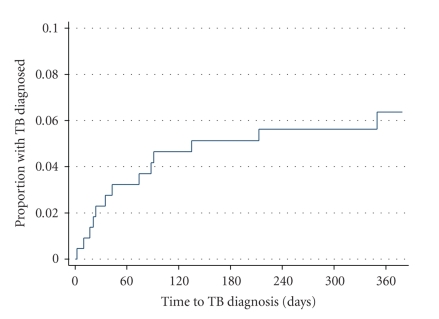
Kaplan Meier curve of cumulative cases of tuberculosis diagnosed among ART naïve patients with HIV infection starting antiretroviral therapy.

Participants diagnosed with TB had a lower BMI and higher levels of CRP protein compared to patients without TB. CRP greater than 5 mg/L and BMI < 18.5 kg/m^2^ were predictive of ART-associated TB in uni- and multivariate analysis (Table 5). When we included patients with unrecognized TB as cases of ART-associated TB, the same factors were also predictive (data not shown). The cumulative cases of all patients diagnosed with TB are shown in Figure 2.

### 3.1. Description of a Patient to Illustrate Unmasking TB-Associated IRIS

**Figure 3:**
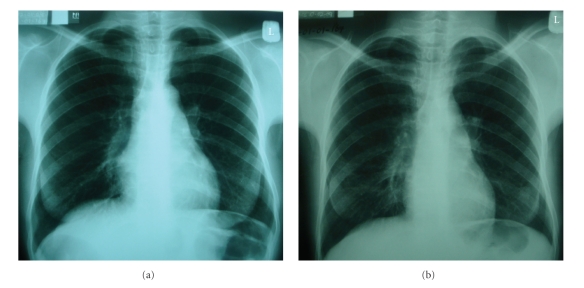


A 25-year-old HIV seropositive female (patient 2, Table 1) started ART (zidovudine/lamivudine/efavirenz) with a CD4 count of 15 cells/*μ*L and viral load 128,056 copies/*μ*L. At ART initiation, she complained of weight loss, reduced appetite, and a skin rash since 2 weeks but had no cough. Physical examination was normal; her BMI was 21.2 kg/m^2^. CXR was normal (Figure 3(a)), but CRP was elevated to 92.21 mg/L. Eleven weeks after starting ART, she developed a productive cough with fever, drenching night sweats, weight loss, and shortness of breath on exertion. On examination, there was moderate abdominal distension due to ascites. Sputum microscopy was AFB + on fluorescence microscopy. A repeat CXR demonstrated bilateral infiltrates in all lung zones (Figure 3(b)). An abdominal ultrasound confirmed the ascites, but there were no abdominal lymphadenopathies. Sputum and ascitic fluid cultures for *M.tb *were positive. At TB diagnosis, the CD4 count was 5 cells/*μ*L and her viral load <400 copies/*μ*L. She fully recovered on completion of TB treatment.

## 4. Discussion

Of 213 patients commencing ART, only 8 (3.8%) developed ART-associated TB. This represents a lower incidence of TB than reported in previous studies and may be due to the systematic strategy used for TB screening: standardized questionnaire for TB-related symptoms, sputum microscopy, and culture, and CXR before commencement of ART. It may also be caused by referral bias as patients with obvious symptoms and signs of TB were not referred. Most previous studies on ART-associated TB and unmasking TB-IRIS have been retrospective [28–30], and few have been able to systematically screen for TB using CXR prior to the start of ART. This may have led to misclassification of prevalent TB disease and over diagnosis of ART-associated TB. Even in our research cohort, there were 6 additional TB cases that were not recognized at study enrolment. An earlier retrospective study performed at the IDI clinic (prior to the INSHI consensus definition) found that 26 (9.6%) of 271 ART naïve patients without a TB diagnosis at onset developed active TB within one year of ART start [31]. Thirty-one percent of these TB patients were diagnosed within 3 months of ART. A more recent study at the IDI clinic found that central nervous system infections and mycobacterial disease were the main causes of HIV-related mortality, especially in the first three months of ART [32]. Our data support systematic intensive screening for TB that includes clinical evaluation, CXRs, sputum microscopy, and culture [13, 14, 33]. Wide-scale implementation of this strategy, however, requires evaluation of cost effectiveness of the methods used and sufficient resources at a programmatic level. 

Only 3 patients in our study developed a clinical picture that could be considered as unmasking TB-associated IRIS according to the INSHI definition (see Table 1). A study performed in South Africa did not find any case of TB-associated IRIS with an intensive pre-ART screening strategy for TB [33]. It is, however, difficult to define TB-associated IRIS in the absence of a biological marker and because of the subjectivity of quantifying the level of inflammatory response.

ART-associated TB represents a heterogeneous group of diseases that develop in the context of starting ART. One form is unmasking TB-associated IRIS which refers to patients without clinical, microbiological, and radiological evidence of TB at onset of ART who develop rapid and exuberant features of inflammation within three months of ART commencement. Another group are patients who were symptomatic before the start of ART but in whom a diagnosis of TB was missed due to lack of sensitive diagnostic tests. Careful followup of such patients with regular evaluation of their symptoms in addition to microbiological and radiological tests may lead to early detection of TB. The third category refers to patients with incident TB who were asymptomatic at the onset of ART but develop signs and symptoms of TB while on ART without the heightened signs and symptoms of inflammation. This may be due to a new infection or a reactivation of latent TB infection. Despite some immunological recovery on ART, patients with HIV infection still have an increased susceptibility to TB infection or reinfection albeit with reduced risk [34].

At multivariate analysis, a raised CRP (5 mg/L or greater) and low BMI (<18.5 kg/m^2^) were predictors of ART-associated TB (Table 3). CRP is a nonspecific direct quantitative measure of acute phase reaction [35, 36]. An increment in the level of CRP is independent of the stage of HIV infection and thus is useful for supporting a diagnosis of opportunistic infections in HIV-coinfected patients. This also makes it useful in monitoring TB treatment response [37]. Low BMI is a marker for poor prognosis in patients with HIV and has also been associated with increased risk of TB and death [38, 39]. Early initiation of TB treatment in HIV-infected patients with wasting and increased CRP levels prior to initiating ART may therefore alleviate the excess morbidity due to undiagnosed TB and ART-associated TB.

The mortality in this cohort of patients was minimal (9(4%) of 219 patients starting ART) compared to other reports. This could be due to the systematic screening and close followup of patients in a study setting with early and effective TB treatment and ART. In particular, TB-IRIS was not a significant cause of early mortality in agreement with retrospective cohort observations [32]. Several studies have demonstrated a survival benefit of early ART in HIV/TB-coinfected patients. In the South African SAPIT trial, a 56% reduction in mortality was observed in patients who started ART during TB treatment compared to those who started after completion of TB treatment [40]. More recently the CAMELIA trial in Cambodia showed that the initiation of ART 2 weeks after starting TB treatment significantly enhanced survival in TB/HIV-coinfected patients compared to starting ART at 8 weeks [41]. This further emphasizes the need to screen for TB prior to ART and to ensure early treatment for both TB and HIV. 

Our study has several limitations. Firstly, the sample size was small. Secondly, 24 patients did not complete the one year followup and 9 of them died. Postmortem examinations were not performed, and therefore we possibly underestimated the burden of TB. Thirdly, in the absence of a biomarker for TB-IRIS, we relied only on clinical evaluation which may be subjective in defining patients with exuberant inflammation. Patients with mild-to-moderate degrees of inflammation were not considered as IRIS. In spite of our efforts to establish an accurate diagnosis of TB, definite diagnosis (sputum smear positivity or culture-positive results) could not be ascertained in 2 patients (patient 1 and patient 8 in Table 4) considered to have ART-associated TB. With sputum induction, bronchoscopy, liquid culture, and/or molecular techniques, more patients with subclinical TB may have been detected. 

In conclusion, careful screening for TB before the start of ART and the continuous assessment of patients for signs and symptoms of TB after starting ART particularly among patients that are wasted and have a raised CRP will lead to an earlier TB diagnosis and ultimately to reduced morbidity and mortality.

## Figures and Tables

**Table 1:** The International Network for the Study of HIV-Associated IRIS (INSHI) case definition for ART-associated tuberculosis [22].

ART-associated tuberculosis	
(i) Patient is not receiving treatment for tuberculosis when ART is initiated.	
(ii) Active tuberculosis is diagnosed after initiation of ART.	
(iii) The diagnosis of tuberculosis should fulfil WHO criteria for smear-positive pulmonary tuberculosis, smear-negative pulmonary tuberculosis, or extrapulmonary tuberculosis.	
Unmasking tuberculosis-associated IRIS*	
(i) Patient is not receiving treatment for tuberculosis when ART is initiated and then presents with tuberculosis within 3 months of starting ART.	
And one of the following criteria must be met:	
(ii) heightened intensity of clinical manifestations, particularly if there is evidence of a marked inflammatory component to the presentation. Examples include tuberculosis lymphadenitis or tuberculosis abscesses with prominent acute inflammatory features, presentation with tuberculosis that is complicated by respiratory failure due to adult respiratory distress syndrome, and those who present with a marked systemic inflammatory syndrome related to tuberculosis,	
(iii) once established on tuberculosis treatment, a clinical course that is complicated by a paradoxical reaction.	
ART: antiretroviral therapy; IRIS: immune reconstitution inflammatory syndrome	

*unmasking tuberculosis-associated IRIS is a subset of ART-associated tuberculosis.

**Table 2:** Baseline characteristics of 219 HIV-infected patients with prevalent TB, incident TB, and no TB.

Characteristics	Prevalent TB (unrecognized) (*n* = 6)	Incident TB (*n* = 8)	No TB (*n* = 205)
Age (yrs), mean (SD)	38.5 (5.3)	38.3 (9.2)	36.4 (8.2)
Gender (Female) (%)	4 (67)	6 (75)	143 (70)
BMI (kg/m^2^), median (IQR)	18.8 (16.6–19.7)*	19.1 (17.9–21.2)**	22.8 (20.4–25.7)
CD4 counts (cell/*μ*L), median (IQR)	131 (60–168)	103 (23–140)	131 (67–172)
Haemoglobin (g/dL), mean (SD)	11.9 (1.9)	10.5 (1.4)**	12.4 (1.9)
C-reactive protein (mg/dL), median (IQR)	24.9 (11.6–31.5)**	19.4 (3.9–51.3)*	2.2 (1.0–6.0)
TST positive at ART start (%)	4 (67)	1 (25)	59 (30)

ART: antiretroviral therapy; BMI, body mass index; IQR: interquartile range; SD: standard deviation; TB: tuberculosis; TST: tuberculin skin test.

*P*-values: *P < .05, **P < .001 compared with the no TB group.

**Table 3:** Characteristics of patients with prevalent (undiagnosed) TB.

Patient	Age (years)	Sex	Baseline CD4 counts (cells/*μ*L)	Baseline clinical symptoms*	Baseline CXR findings	Time to TB diagnosis (days)	TB category	Basis of TB diagnosis	Outcomes
Patient 1	32	F	6	cough, chest pain, weight loss, poor appetite	middle and lower lung zone infiltrates	24	PTB	culture+, worsening CXR	improved
Patient 2	43	M	168	no symptoms	upper and middle lung zone infiltrates with cavitation	35	PTB	worsening CXR	improved
Patient 3	35	F	121	cough, fever, weight loss, poor appetite	normal	65	PTB	culture+	died
Patient 4	46	F	60	cough, chest pain, poor appetite	upper lung zone infiltrates and left side pleural effusion	0	EPTB	abnormal CXR	improved
Patient 5	35	F	140	cough	middle and lower lung zone infiltrates and pleural thickening	43	PTB	abnormal CXR	lost contact
Patient 6	41	M	180	no symptoms	middle and lower lung zone infiltrates and bilateral cavities	10	PTB	abnormal CXR	improved

Abbreviations: M: Male; F: Female; TB: tuberculosis; CXR: chest X-ray; PTB, pulmonary tuberculosis, AFB+: acid fast bacilli positive; AFB−: acid fast bacilli negative; IRIS: immune reconstitution inflammatory syndrome.

*included only clinical symptoms of greater than two weeks.

**Table 4:** Characteristics of patients with ART-associated TB.

Patient	Age (years)	Sex	CD4 counts		Baseline clinical symptoms*	Baseline CXR findings	Time to TB (days)	TB category	Basis of TB diagnosis	Basis of TB- IRIS diagnosis^†^	Outcomes
			Baseline (cell/*μ*L)	TB (cell/*μ*L)							
Patient 1	53	F	156	148	weight loss	Possible TB	345	TB pleura	cytology	NA	Improved
Patient 2	27	F	15	5	weight loss, anorexia	Normal	79	Disseminated TB	abnormal CXR culture +ve	2,4,5,6	Improved
Patient 3	31	F	90	68	cough, fever, weight loss, anorexia	Normal	14	PTB	culture +ve	NA	Improved
Patient 4	37	M	123	—	no symptoms	Normal	24	PTB	culture +ve	NA	Lost contact
Patient 5	42	M	116	95	cough, weight loss, anorexia	Possible TB	54	PTB	culture +ve	NA	Died
Patient 6	45	F	21	28	weight loss	Normal	17	PTB	AFB+	2,5,6,7	Improved
Patient 7	28	F	24	18	no symptoms	Normal	20	TB adenitis	FNA AFB+	1,5,6	Lost contact
Patient 8	43	F	206	313	no symptoms	Normal	100	PTB	abnormal CXR	NA	Improved

Abbreviations: M: Male; F: Female; TB: tuberculosis; CXR: chest X-ray; PTB: pulmonary tuberculosis; AFB+: acid fast bacilli positive; AFB−: acid fast bacilli negative; IRIS: immune reconstitution inflammatory syndrome.

*included clinical symptoms of greater than two weeks.

^†^criteria for TB-IRIS (new or worsening): 1: adenopathy; 2: CXR abnormalities; 3: central nervous system features of TB; 4: serositis; 5: constitutional symptoms (fever, night sweats, weight loss); 6: respiratory symptoms (cough, dyspnea, stridor); 7: abdominal pain with peritonitis, hepatomegaly, splenomegaly, or adenopathy; NA: not applicable.

**Table 5:** Cox proportional hazards for baseline predictors of ART-associated TB in HIV-infected patients commencing ART.

Baseline characteristics	Unadjusted HR (95% CI)	*P*-value	Adjusted† HR (95% CI)	*P*-value
Age (years)				
>40	1.42 (0.26–7.77)	.687	1.27 (0.21–7.80)	.798
30–39	0.43 (0.06–3.05)	.397	0.35 (0.05–2.64)	.308
<30	1		1	
Sex				
Females	1.19 (0.24–5.88)	.835	1.87 (0.34–10.21)	.468
Males	1		1	
Tuberculin skin test (TST)				
positive	0.78 (0.16–3.88)	.764	—	
negative	1		—	
C-reactive protein (mg/L)				
≥5	8.23 (1.66–40.82)	**.010**	7.23 (1.36–38.33)	**.020**
<5	1		1	
Haemoglobin (g/dL)				
<12.5	6.66 (0.82–54.16)	.076	2.31 (0.23–23.43)	.477
≥12.5	1		1	
CD4 cell counts (cells/*μ*L)				
<50	3.04 (0.72–12.74)	.129	2.22 (0.49–10.16)	.304
≥50	1		1	
Body mass index (kg/m^2^)				
<18.5	7.71 (1.92–30.89)	**.004**	5.85 (1.24–27.46)	**.025**
≥18.5	1		1	
WHO clinical stage				
3 and 4	4.68 (0.94–23.23)	.065	1.82 (0.33–10.18)	.495
1 and 2	1		1	

†adjusted for age, sex, baseline C-reactive protein result, baseline haemoglobin, baseline CD4 counts, body mass index, and WHO clinical stage.

Abbreviations: ART: antiretroviral therapy; TB: tuberculosis; HR: hazard ratio.
